# Assessment of the Occupational Risk of Tuberculosis & Air Borne Infection Control in High-Risk Hospital Wards and Its Implications on Healthcare Workers in a Tertiary Care Hospital in South India

**DOI:** 10.7759/cureus.33785

**Published:** 2023-01-15

**Authors:** Syed Abdul Bari, Qursheed Sultana, Qader A Jalily, Mummareddi Dinesh Eshwar, Saikrishna Dodda

**Affiliations:** 1 Microbiology, Mahavir Institute of Medical Sciences, Hyderabad, IND; 2 Microbiology, Dr. Vizarath Rasool Khan (VRK) Women's Medical College, Hyderabad, IND; 3 Medicine, Navodaya Medical College, Raichur, IND

**Keywords:** airborne infection control, microbiological air sampler, occupational diseases, healthcare workers, tuberculosis, respiratory intensive care units, hospitals

## Abstract

Introduction

The indoor air in hospitals could play a significant role in the transmission of a wide array of infections, especially in respiratory intensive care units, pulmonary outpatient departments, and other areas. Unprotected coughing and sneezing may facilitate the release of aerosols and contaminate the indoor environment. The majority of infections transmitted through these modes include viral diseases, including tuberculosis (TB), influenza, and measles, among several others. Moreover, the possibility of direct and indirect transmission of microbes by air has been underestimated in hospital settings, especially in developing countries. This study therefore was carried out to assess the burden of microbes in the air of selected wards in a tertiary care hospital and evaluate the occupational risk of some infections among healthcare workers (HCWs).

Methods

This study was carried out between September 2019 and February 2021 at a tertiary care teaching hospital in South India. A total of 30 symptomatic healthcare workers (HCWs) were included in the study and were screened for present and past tuberculosis (TB) as well as other lower respiratory tract infections. A tuberculin skin test, chest X-ray, and sputum acid-fast staining were performed on all the HCWs who were negative for other bacterial infections and were symptomatic. The study was conducted in coordination with the pulmonology department. Active monitoring of air was performed by microbiological air sampler in the respiratory intensive care unit (RICU) and other high-risk areas including the pulmonology outpatient department (OPD), the radiology OPD, and the microbiology department.

Results

Sputum for tuberculous bacteria was positive in four (16.6%) HCWs. The chest X-ray showed radiological findings suggestive of TB in five (20.8%) HCWs. Three (12.5%) HCWs who were screened for extrapulmonary TB revealed one (33.3%) was positive for TB of the hip joint. Among the HCWs, eight (33%) returned positive tuberculin tests. Assessment of the hospital air in the RICU revealed the bacterial count (288 CFU/m^3^) exceeded the normal limit (≤50 CFU/m^3^). The COVID-19 isolation ward showed the lowest bacterial count (06 CFU/m^3^) and no fungi. The predominant bacterial isolates were gram-positive cocci in clusters (Methicillin-sensitive *Staphylococcus aureus*). After proper disinfection and correction of ventilation techniques, the resampling results noted microbial colonies under normal limits.

Conclusion

A high burden of TB was noted among the HCWs. The airborne infection control strategies are essential to minimize the risk of nosocomial infections and occupational TB risk to HCWs. Most microbes are transmitted through the airborne route and therefore it is extremely important to take measures to control the transmission of such pathogens in hospital settings.

## Introduction

The hospital atmosphere plays an influential role in causing nosocomial infections. This is because the hospital environment comprises a variety of microbes [1]. Common bacterial species associated with healthcare-associated infections include *Staphylococcus aureus*, *Pseudomonas aeruginosa*, *Escherichia coli*, enterococci, *Acinetobacter *spp., and Coagulase-negative staphylococci [2-5]. Moreover, the majority of the bacterial isolates were identified as resistant to multiple antibiotics (multi-drug resistant) [5]. These microbes can endure and prevail for a long duration of time in the hospital environment and are not affected by chemical disinfection [6]. The bacterial pathogens with their ability to survive in the hospital environment could be responsible for various types of nosocomial infections [5]. Transmission of airborne diseases caused by the influenza virus, mycobacteria, coronaviruses, and Nipah virus, among others, have been previously reported [7,8]. Hospital transmission of infections is linked to limited airborne infection control strategies [9].

Proper ventilation, especially in closed rooms without windows is critical to controlling respiratory diseases. Hospital building ventilation (both natural and mechanical ventilation) has vital elements that include ventilation rate wherein the portion of outdoor air that can be housed in the space, the quality of the outdoor air, and airflow movement wherein the overall airflow direction in the facility moves from hygienic zones to unclean zones. Also important is the air dispersal or airflow pattern wherein the external air should be reaching each part of the space in an efficient way and the airborne contaminants present in each region of the area should also be removed in an efficient way [10]. Moreover, hospital air could pose risk for nosocomial infections and occupational diseases among patients and healthcare workers (HCWs), respectively.

This study is therefore carried out to assess the microbiological quality of hospital air and evaluate the burden of occupational diseases with special reference to tuberculosis among HCWs in a tertiary care hospital in South India.

## Materials and methods

A total of 30 HCWs who had symptoms of lower respiratory tract infections were included in the study. This study was carried out between September 2019 and February 2021 at a tertiary care teaching hospital in South India. The participants included in the study had symptoms such as cough for more than two weeks, fever, weight loss, breathlessness, and arthralgia. A sputum sample was collected from each study participant and was processed for routine culture. Four subjects revealed the growth of bacterial pathogens and were excluded from the study. Among the remaining 26 participants, two were unwilling to continue in the study and were excluded. The study, therefore, was continued with the remaining 24 HCWs who were screened for present and past tuberculosis.

Active monitoring of air was performed by microbiological air sampler in the respiratory intensive care unit (RICU) and other high-risk areas including the pulmonology outpatient department (OPD), the radiology OPD, and the microbiology department. The study was approved by the Institutional ethics committee (approval no.: 2019/27/001) and informed consent was obtained from all the study participants. 

Assessment of occupational diseases (tuberculosis) among symptomatic HCWs

A tuberculin skin test, chest X-ray, and sputum acid-fast staining were performed on all 24 HCWs. Of the 24 suspected cases of TB, three HCWs complained of arthralgia. On clinical and radiological examination, a provisional diagnosis of bone TB was made and the joint fluid was tested for the presence of acid-fast bacilli.

Evaluation of the quality of air

**Figure 1:**
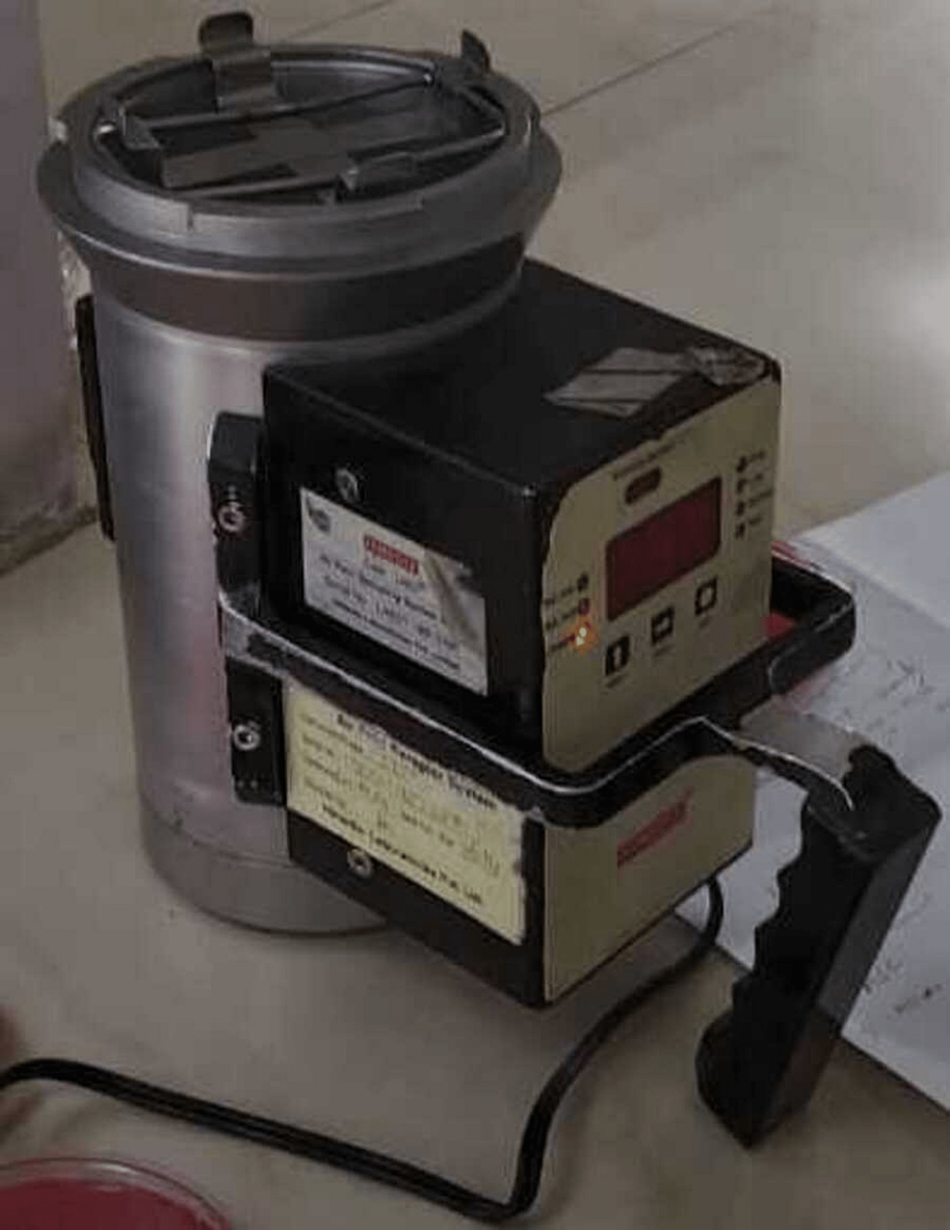
The microbiological air sampler

The active monitoring of air was carried out with the microbiological air sampler (Bioaerosol sampler manufactured by PBI International) as shown in Figure 1.

**Figure 2:**
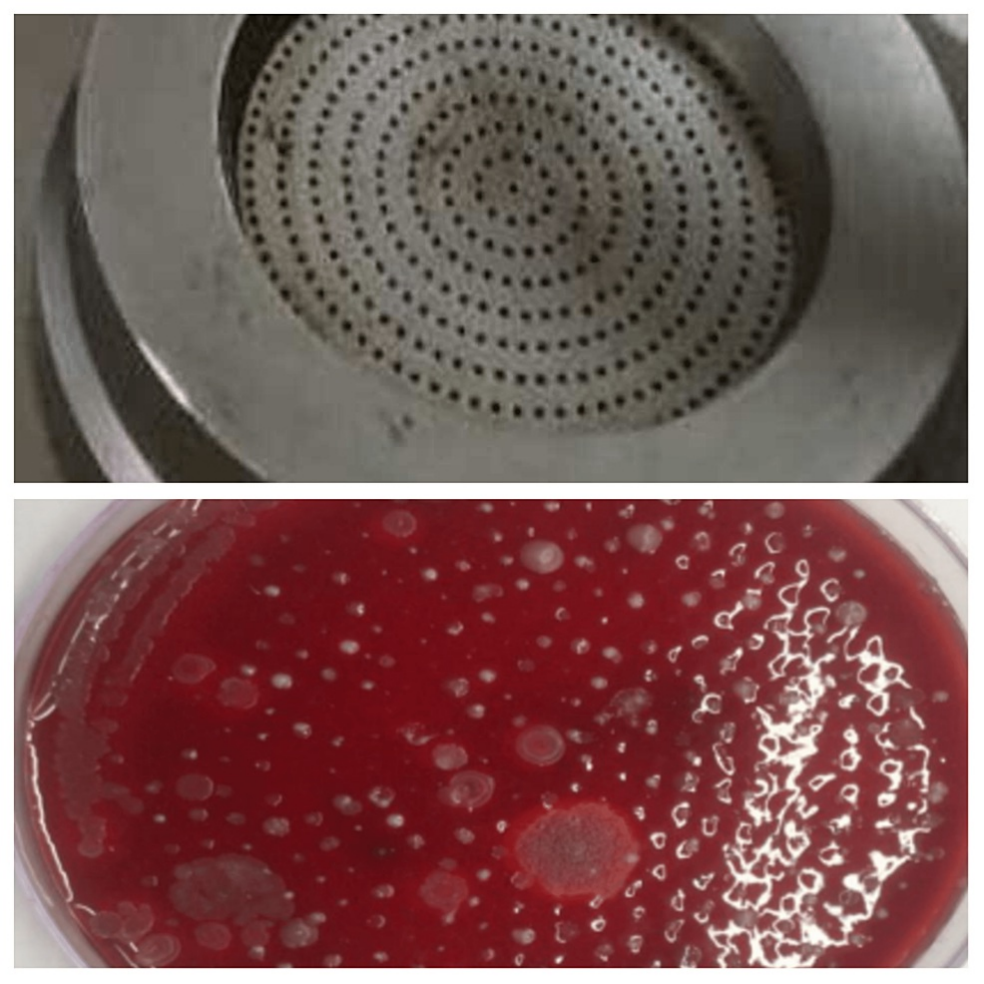
Microbial colonies as impinged by the air sampler

This instrument physically draws a known volume of air through a particle collection device which is later inoculated on solid media. The instrument consists of a vacuum pump container that is designed to accommodate a petri dish. In our study, we used blood agar plates of 90 mm diameter. The cover on the unit is perforated with sieve-like perforations of a predetermined size. A vacuum pump draws a 1000 L volume of air through the cover for about 10 minutes and the particles in the air containing microorganisms impinged on the blood agar plate. After the collection cycle, the petri dish is incubated for 18 to 24 hours at 37^0^C in an incubator. The colonies are counted for and expressed as colony-forming units (CFU) per/m^3 ^(Figure 2).

The colonies counted on the plate do not reflect the actual organism load in the air. This is because the number of viable particles being impinged on a given plate increases the probability of the next particle going into an empty hole decreases. Therefore, the number of CFU counted on the petri dish needs a statistical correction by using a formula: Pr =N (1/N+1/N-1+1/N-2…….1/N-r+1), where Pr=probable statistical total; r=number of CFU counted on 90 mm petri dish; N=total number of holes. This formula estimates the actual number of organisms per cubic meter of air sampled. 

The study followed the guidelines framed by the Scottish quality assurance specialist interest group and the essentials of hospital infection control practices as suggested by Sastry et al. [11,12].

## Results

Sputum for tuberculous bacteria was positive in four (16.6%) HCWs. The chest X-ray showed radiological findings suggestive of TB in five (20.8%) HCWs. Three (12.5%) HCWs who were screened for extrapulmonary TB revealed one (33.3%) was positive for TB of the hip joint and also revealed a chest X-ray suggestive of TB. Among the 24 HCWs, eight (33%) returned positive tuberculin tests. Those positive for the tuberculin test were referred to pulmonology OPD for further evaluation. In 30 HCWs that included clinicians and microbiologists, the routine bacterial cultures revealed four (13.3%) positives. Out of the four positive cases, two HCWs showed *Klebsiella pneumoniae *and one each revealed methicillin-sensitive *Staphylococcus aureus *and *Streptococcus *species. The details of the evaluation of HCWs for tuberculosis and other bacterial infections are presented in Table 1.

**Table 1:** Evaluation of healthcare workers for tuberculosis and other bacterial infections HCWs: Healthcare workers; AFB: Acid-fast bacilli

Name of test	Total HCWs screened	Positives	Interpretation
Sputum for AFB	24	04	Suggestive of pulmonary tuberculosis
Chest X-ray	24	05	Suggestive of pulmonary tuberculosis
Body fluid for AFB	03	01	Suggestive of hip joint/extrapulmonary tuberculosis
Tuberculin skin test	24	08	Suggestive of latent tuberculosis
Sputum for routine culture	30	04	Suggestive of bacterial lower respiratory tract infection

Active monitoring of air by the microbiological air sampler in various wards of the hospital revealed that the RICU had the highest bacterial count (288 CFU/m^3^) and exceeded the normal limit (≤50 CFU/m^3^). The COVID-19 isolation ward revealed the lowest bacterial count (06 CFU/m^3^). The results of the air quality of different sections of the hospital are outlined in Table 2.

**Table 2:** Microbiological assessment of air in different wards of the hospital Pr: Probable statistical total; ICU: Intensive care unit; OPD: Outpatient department; CT: Computed tomography

Area of air sampling	Number of bacterial colonies isolated (Pr)	Bacteria isolated on culture	Number of fungal colonies isolated	Normal count of bacterial colonies in designated areas	Normal count of fungal colonies
Respiratory ICU	288	Staphylococcus aureus	01	≤50	≤5
Pulmonology OPD	23	Staphylococcus aureus	02	≤35	Nil
COVID-19 isolation room	06	*Staphylococcus aureus* and a few Gram-negative bacilli	Nil	≤35	Nil
Corridor	12	Micrococcus	Nil	≤35	Nil
Cardiac ICU	14	Staphylococcus aureus	Nil	≤35	Nil
Surgical ICU	12	Coagulase-negative *Staphylococcus* (CONS)	Nil	≤35	Nil
CT-scan room	07	Staphylococcus aureus	Nil	≤35	Nil
Ultrasound room	33	Staphylococcus aureus	Nil	≤35	Nil
Bacterial culture room (Microbiology)	23	Staphylococcus aureus	Nil	≤35	Nil

## Discussion

Respiratory infections are potentially transmitted airborne and are caused by different microbes that include bacteria, viruses, and fungal species. Such infectious diseases pose a significant risk for patients and an occupational hazard for HCWs. The risk of infection is further heightened with deficient infection control procedures [13]. The current study demonstrates the evaluation and the measures needed to control airborne infectious diseases. In our study, we screened HCWs working in high-risk areas such as clinical laboratories and the pulmonology department for any respiratory illness like chronic cough (> 2 weeks), fever, weight loss, and breathlessness. The clinical evaluation was done among study participants based on their clinical history, physical examination, and laboratory evaluation. The overall incidence of TB in our study was 20.8% (5/24), which is in correlation with the study conducted by Aggarwal et al. who reported an overall incidence of 17.3 per 1000 [14]. A higher incidence of TB in our study could be due to the small study group and evaluation of only high-risk and symptomatic HCWs.

Tuberculosis is extremely prevalent in India and it poses an increased risk both to common people and HCWs. Since patients suspected to be suffering from TB present to the hospitals for care and because HCWs are involved in the management of TB patients, the HCWs can potentially be exposed to and suffer from TB. Therefore, it is important to collect useful data that are essential for notifying the implementation of TB infection control measures [15]. Because TB is transmitted through respiratory droplets, enforcing respiratory infection control standards could contribute to minimizing the transmission. Effective airborne infection control measures prevent new infections, especially among HCWs [15]. Hospital administrators could play a key role in identifying the risks of TB and implementing infection control measures. Moreover, hospital administrators may carry out regular evaluations of HCWs for TB. Such an evaluation should be carefully carried out on a semi-annual or annual basis and appropriately documented [15]. The result of active air surveillance in our study showed high colony count in RICU than in other critical areas and the majority of isolates were gram-positive cocci in clusters (*Staphylococcus aureus *and others) which is in correlation with a study by Banerjee et al. from India wherein methicillin-sensitive *Staphylococcus aureus* (MSSA) was predominant [16]. Despite these bacteria being transmitted majorly through contact and fomites, their presence warrant improved infection control measures on the hospital premises.

In poor and developing countries like India, the healthcare facilities are insufficient and therefore HCWs are vulnerable to infections. India accounts for more than a quarter of the global burden of TB and is densely populated providing an easy opportunity for transmission that necessitates better infection control practices [17]. Unfortunately, the TB and infection prevention control based on national and international guideline implementation is currently unsatisfactory. Moreover, nosocomial transmission has been identified as a significant method of transmission of TB and drug-resistant TB [18]. Interestingly, studies concerning the occupational risk of TB and other respiratory illnesses have been grossly ignored. 

Study limitations

This study had several limitations including a small sample size and a single-center study. Only those attending the RICUs, pulmonology department, and microbiology laboratory were included in the study. Moreover, only bacterial and fungal species were evaluated and the viral etiology was not addressed both in the HCWs as well as in the hospital environment. Molecular methods for screening microbial species were not employed. A limited number of HCWs were screened for respiratory infections including TB. Moreover, this is just a point prevalence study and therefore the results generated from this study may be not suitable for generalization purposes.

## Conclusions

The results of this study identified shortcomings in compliance with the implementation of certain components of national and international policies/guidelines in high-risk areas of hospital environments. Along with the environmental factors, patients and healthcare workers have equal contributions to microbial dispersion and cross-transmission of infection in the ICUs. Improvement of the ventilation system, environmental conditions, restricted human movement, strict barrier precautions, and installation of high-efficiency particulate air filters will help in decreasing the infection rate in the ICUs. Guidelines for the patient-to-healthcare workers' ratio require careful evaluation and need to be strictly maintained. This study's results emphasize the need for modification in the air ventilation system, restricted entry of patient attendants and other medical staff, and fumigation of high-risk areas as evidenced by the observation of microbial counts that were under normal limits post disinfection. The systematic scale-up of airborne infection control measures across all healthcare facilities may ensure a readiness plan to prevent any future airborne infections of pandemic potentials in HCWs as being experienced with the current COVID-19 pandemic.
